# Reconstructed Ancestral *Myo*-Inositol-3-Phosphate Synthases Indicate That Ancestors of the *Thermococcales* and *Thermotoga* Species Were More Thermophilic than Their Descendants

**DOI:** 10.1371/journal.pone.0084300

**Published:** 2013-12-31

**Authors:** Nicholas C. Butzin, Pascal Lapierre, Anna G. Green, Kristen S. Swithers, J. Peter Gogarten, Kenneth M. Noll

**Affiliations:** Department of Molecular and Cell Biology, University of Connecticut, Storrs, Connecticut, United States of America; University of North Dakota School of Medicine and Health Sciences, United States of America

## Abstract

The bacterial genomes of *Thermotoga* species show evidence of significant interdomain horizontal gene transfer from the Archaea. Members of this genus acquired many genes from the Thermococcales, which grow at higher temperatures than *Thermotoga* species. In order to study the functional history of an interdomain horizontally acquired gene we used ancestral sequence reconstruction to examine the thermal characteristics of reconstructed ancestral proteins of the *Thermotoga* lineage and its archaeal donors. Several ancestral sequence reconstruction methods were used to determine the possible sequences of the ancestral *Thermotoga* and Archaea *myo*-inositol-3-phosphate synthase (MIPS). These sequences were predicted to be more thermostable than the extant proteins using an established sequence composition method. We verified these computational predictions by measuring the activities and thermostabilities of purified proteins from the *Thermotoga* and the Thermococcales species, and eight ancestral reconstructed proteins. We found that the ancestral proteins from both the archaeal donor and the *Thermotoga* most recent common ancestor recipient were more thermostable than their descendants. We show that there is a correlation between the thermostability of MIPS protein and the optimal growth temperature (OGT) of its host, which suggests that the OGT of the ancestors of these species of Archaea and the *Thermotoga* grew at higher OGTs than their descendants.

## Introduction

From the publication of the first genome sequence of a member of the bacterial order Thermotogales, that of *Thermotoga* (*Tt.*) *maritima*; the importance of gene sharing with the Archaea was apparent for this lineage [1]. Subsequent examinations of the genomes from the other Thermotogales showed that these organisms have extensively shared genes with the Archaea through horizontal gene transfer (HGT) [2]. Many of these archaeal genes were derived from the Thermococcales, mostly represented in modern species as hyperthermophiles with optimal growth temperatures (OGTs) above those of Thermotogales species. Consequently, the genes inherited by the Thermotogales likely encoded proteins catalytically active at temperatures higher than that at which modern Thermotogales grow. The ancestral Thermotogales that inherited these genes likely grew at temperatures higher than modern species and so was suited to use them [2]. Genes acquired from the Thermococcales are largely found in the *Thermotoga* species, the species of Thermotogales with the highest OGTs. Thus, to examine the evolution of proteins acquired from the Archaea, we chose to reconstruct an ancestral protein, a *myo*-inositol-3-phosphate synthase (MIPS), shown to have been acquired by *Thermotoga* species from the Archaea (Nesbo et al. 2001).

MIPS is an essential enzyme in the *Tt. maritima* biosynthetic pathway for the compatible solute di-myo-inositol-1,1-phosphate [3]. MIPS is common in both euryarchaeotes and crenarchaeotes, but is rarely found in Bacteria [4]. There is strong support that the MIPS gene and other genes, which allow for synthesis of *myo*-inositol phosphate, originated in an archaeal lineage [4]–[7]. *Tt. maritima* accumulates several types of inositols at superoptimal temperatures [8]. Recently, Gonçalves and co-workers showed that the *Thermotoga* ancestor acquired several genes for synthesis of various inositols [4]. They also determined that the enzymes used to synthesize *myo*-inositol-phosphate are limited to Bacteria and Archaea with OGTs above 55°C [4]. The importance of inositols for growth at high temperatures underscores the selective advantage that *Thermotoga* may have gained by acquiring a MIPS gene from the euryarchaeota.

To trace the functional history of the *Thermotoga* MIPS, we used ancestral sequence reconstruction (ASR) to reconstruct ancestral proteins at specific nodes of the MIPS phylogenetic tree. ASR predicts the sequences of ancestral proteins based on those of their modern homologs using phylogenetic relationships and statistical approaches [9]–[11]. Aligned sequences are used to predict the most probable amino acid residues in ancestral proteins, which can then be reconstructed by synthesizing the predicted genes and expressing recombinant proteins in a suitable host [9]–[11]. In previous studies ASR was used to predict properties of ancient life by analyzing reconstructed proteins from Eukaryotes [12]–[14], Archaea [15], and Bacteria [9], [10], [15], [16].

Since ASR has an established record of predicting properties of ancient life, here we use it to test the hypothesis that the ancestral *Thermotoga* MIPS is more thermal stable and catalytically active at a higher temperature than extant *Thermotoga* MIPS proteins. In addition, we tested the related hypothesis that reconstructed archaeal MIPS proteins are also active and stable at higher temperatures than their modern descendants. To test these hypotheses we reconstructed ancestral proteins for both the *Thermotoga* and the Archaea nodes of the MIPS phylogenetic tree and examined their biochemical properties. We compared these with other MIPS proteins derived from extant species. The results of these tests are consistent with the hypothesis that the ancestors of the *Thermotoga* and the Thermococcales species were higher temperature hyperthermophiles.

## Results and Discussion

### Thermotoga Myo-inositol-3-phosphate Synthase (MIPS) was Inherited from Archaea

In order to examine the characteristics of ancestral proteins inherited by interdomain HGT, the *Thermotoga* genomes were screened for a gene of archaeal origin whose protein product met the following criteria. First, only soluble proteins were considered to avoid the potential difficulties associated with membrane protein expression, purification, and solubilization. Second, enzymes with easily measured activities were sought to allow facile functional characterization of the proteins’ activities. Third, only small, monomeric enzymes were acceptable so that only a single gene need be constructed. Fourth, proteins with known crystal structures were desirable to identify residues important for activity and to guide selection of amino acids important for catalysis. Finally, proteins were only considered if they had a clear phylogenetic signal indicating horizontal inheritance from Archaea. Based on these criteria, we chose to examine the thermal characteristics of *Thermotoga myo*-inositol-3-phosphate synthase (MIPS; EC 5.5.1.4) [17], a protein that shows a clear phylogenetic origin in the Archaea.

**Figure 1:**
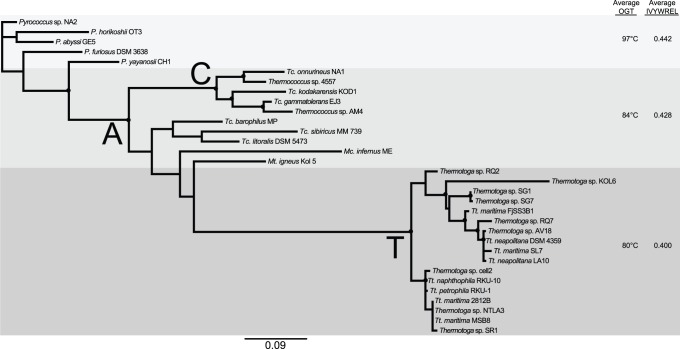
Maximum likelihood (PhyML) tree of *Thermotoga* MIPS homologs. The tree was constructed using PhyML and support values were calculated from 100 bootstrap samples. Sequences were from *Thermotoga* (*Tt.*), *Thermococcus* (*Tc.*), *Pyrococcus* (*P.*), *Methanocaldococcus* (*Mc.*), and *Methanotorris* (*Mt.*). The average OGT and IVYWREL bias values (IVYWREL) were determined for each shaded group. Black filled circles (•) indicate ≥70 bootstrap support. Nodes T (the ancestor of all *Thermotoga* species), C (the ancestor of a group of *Thermococcus*), and A (ancestral to both node T and C and to other Archaea) have 100 bootstrap supports. The *P. horikoshii* OT3 MIPS sequence (gi 14591380) was modified from that in the NCBI database by removal of amino acids 1–42 because they did not show homology to any other MIPSs suggesting a possible misannotation of its start codon.

A previous study showed that the *Thermotoga* genus acquired a MIPS gene from an archaeal lineage [18]. The number of sequenced genomes has grown significantly since that work was published and more sequenced MIPS genes are available (Table S1), which provided an opportunity to reexamine these relationships. An updated phylogenetic analysis of *Thermotoga* MIPSs revealed further support for its origin in the Archaea, specifically among the Euryarchaeota (Figure S1). The distribution of the MIPS protein within the Thermotogales is unique to the *Thermotoga* genus. Together the phylogeny and the distribution of the MIPS gene within the Thermotogales strongly support that the MIPS gene was transferred to the *Thermotoga* ancestor, rather than transferred to the common ancestor of all Thermotogales and then independently lost in the other lineages (Figure 1 and S1).

### Prediction of Ancestral *Thermotoga* and Archaea MIPS Proteins

Ancestral sequence reconstruction (ASR) was used to reconstruct ancestral proteins at specific nodes of the MIPS phylogenetic tree. Several different methods of ASR were compared to determine which method resulted in minimal apparent biases for the MIPS dataset. The ancestral sequences were predicted using Ancescon [19] with a maximum likelihood (ML) tree calculated using PhyML as reference for the reconstruction. Ancestral proteins were predicted for three strongly supported nodes, the most recent common ancestor (MRCA) to the *Thermotoga* (node T), the MRCA to the *Thermococcus* node (node C), and the node where nodes T and C and other Archaea (node A) branches join (Figure 1). One potential problem with Ancescon is that it assumes a homogeneous amino acid composition throughout the tree, which may produce ancestral proteins with amino acid compositions artificially biased to those of the extant proteins.

This bias was examined by generating the ancestral proteins using BppAncestor [20] and a non-homogeneous substitution model that allowed for a separate set of parameters for each clade. The predicted ancestral sequences using BppAncestor (non-homogeneous substitution model) and Ancescon (homogeneous) were quite similar and only varied slightly. There is no significant difference between the BppAncestor (non-homogeneous substitution model) and the final four reconstruct sequences (ATM_T1-4; explained below) IVWREL value (see next section for correlations) with means of 0.4085 and 0.4110, respectively (*p* = 0.998; *t*-test). This shows there is no composition bias toward the extant sequences when the Ancescon is used on our dataset; thus, the homogeneous model was used in further analyses.

Another potential problem stemming from a simple ML approach to ASR is it may lead to a bias towards more thermostable proteins for some datasets [21]. We employed a Bayesian method that samples from a posterior distribution to estimate the ancestral amino acid composition. Such a method is not prone to the tendency of ML methods to reconstruct ancestral proteins with a higher thermostability [21]. Ancestral sequences were predicted using BppAncestor with both the non-homogeneous model described above, and a homogeneous implementation of that same model. For each ancestral sequence, 1000 replicates were determined by sampling from the posterior distribution. As a quantitative marker of thermostability, we used the total fraction of seven amino acids (IVYWREL) for each sequence [22] (see next section for correlations). The mean and confidence interval of the IVYWREL values for the ancestral sequences were calculated from these replicates (Table S2). The IVYWREL values for the ML based ancestral reconstruction are within a 95% confidence interval determined through sampling from the posterior probabilities, indicating that our dataset is not prone to the bias mentioned by [21]. This method results in a large number of potential reconstructed proteins making it impractical to produce proteins for biochemical analysis. However, our results suggest that the ML approach applied to this dataset does not have a strong basis towards a more thermostable ancestral reconstruction.

Taken all together these analyses show that Ancescon does not introduce an apparent bias towards generating thermostable proteins, nor does it introduce compositional biases to the extant sequences for our dataset. To account for variance in tree topology, (nodes with <70 bootstraps were considered to be weakly supported, Figure 1), Ancescon was used for ASR of sequences at nodes A, C, and T (Figure 1) generated from 1000 Bayesian trees (See Materials and Methods). These analyses resulted in four probable ancestor proteins predicted at node T, called ancestral *Thermotoga* MIPS, ATM (ATM_T1-4). The reconstructed proteins for node C were called ancestral *Thermococcus* MIPS, ACM (ACM_C1-2), and the reconstructed proteins for node A were called ancestral Archaea MIPS, AAM (AAM_A1-2).

### *In silico* Analyses of MIPSs Show that the Ancestral *Thermotoga* MIPSs are more Thermostable

A genome scale correlation has been shown between the OGT of an organism and the total fraction of seven amino acids (IVYWREL) in its soluble proteins [22]. To determine if this relationship holds for a single protein family a large sampling of MIPS protein IVYWREL values were compared to their respective organisms’ OGTs (Figure S1). The comparison of MIPS proteins from Figure S1 (Pearson correlation coefficient [Pcc] = 0.80; Figure S2) resulted in a similar pattern as observed previously at the genome scale. This suggested that IVYWREL values can be used as an indication of thermostability of MIPS proteins (Figure S2). Additionally the OGT of the organisms in Figure 1 are correlated to their MIPS IVYWREL (R^2^ = 0.85; Figure S3A). This pattern suggests that the previous correlation observed between OGT and IVYWREL on a genome scale holds for the smaller set of MIPS proteins as well, and that IVYWREL can be used as a general indicator of protein thermostability.

IVYWREL values were calculated for all reconstructed ancestral proteins. ATM_T1-4 all have higher IVYWREL values than the average of the extant *Thermotoga* MIPSs (*p*<0.001, Table S3; Figure 1; Table 1) suggesting greater thermostability of the ancestral *Thermotoga* MIPSs than modern *Thermotoga* MIPSs. Sequences ACM_C1-2 have similar IVYWREL values as the extant *Thermococcus* MIPSs of node C (*p*>0.7). AAM_A1-2 sequences are all higher in their IVYWREL values than that of sequences from Node T and Node C (Figure 1), and ATM_T1-4 (*p*<0.001; Table S3).

**Table 1:** Measures of thermostability of extant and ancestral MIPSs.

Organism	Protein	OGT (°C)	T_opt_ (°C)	T_m_ (°C)			IVYWREL value
				pH 7.0	pH 4.2	pH 3.5	
*Tc. sibiricus* MM 739	TSIB_1788	78	ND	81.1±0.06	63.5±0.20	ND	0.4184
*Tt. maritima* MSB8	TM1419	80	75	81.0±0.16	64.5±0.17	ND	0.4031
*Thermotoga* sp. str. RQ2	TRQ2_1313	80	80	85.2±0.07	63.6±0.14	ND	0.4005
	ATM_T1		90	88.6±0.05	66.7±0.09	44.7±0.47	0.4084
	ATM_T2		83	88.8±0.02	67.5±0.09	47.9±0.07	0.4110
	ATM_T3		85	88.9±0.08	68.1±0.17	47.4±0.13	0.4110
	ATM_T4		83	88.9±0.01	68.3±0.39	49.2±0.13	0.4136
	ACM_C1		95	>99	76.6±0.11	55.2±0.11	0.4188
	ACM_C2		95	>99	76.5±0.04	55.3±0.03	0.4162
*Tc. kodakarensis* KOD1	TK2278	85	95	>99	80.2±0.12	57.7±0.23	0.4215
*P. furiosus* DSM 3638	PF1616	100	99	>99	81.6±0.08	69.1±0.15	0.4334
	AAM_A1		99	>99	>99	89.7±0.25	0.4491
	AAM_A2		99	>99	>99	90.6±0.17	0.4465

The *T_m_* values of MIPSs were determined using DSF. The *T_opt_* values were determined using a MIPS/malachite green assay. Standard deviations for MIPS *T_m_* values were determined from three replicates. *T_opt_* values were determined from at least four replicates. ND, not determined.

The *in silico* analyses suggests that the *Thermotoga* and *Thermococcus* ancestors may have been capable of living in hotter environments than their descendants do now. This contention is based on the fact that the reconstructed proteins at each node are predicted to be more thermostable than their descendant proteins. Since the thermal characteristics of extant MIPS proteins show a correlation with the OGTs of their hosts, we surmise that the hosts of our reconstructed ancestral proteins had OGTs higher than their descendants.

**Figure 2:**
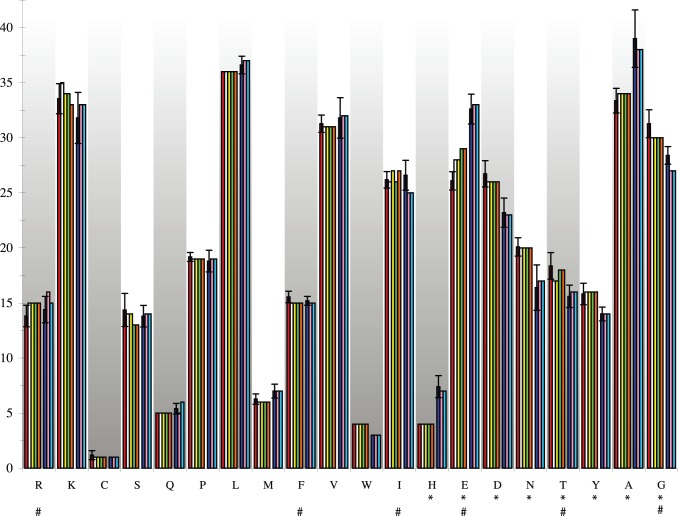
Comparison of amino acid compositions between *Thermococcus* sequences and *Thermotoga* sequences. The y-axis is amino acid (AA) counts and the x-axis is the amino acid. Red bars are the mean of the extant *Thermotoga* sequences; white, yellow, green and orange bars are the *Thermotoga* ancestral reconstructed sequences ATM_T1–T4, respectively. Dark blue bars are the mean archaeal sequences, and pink and light blue bars are the *Thermococcus* ancestral reconstructed sequences ACM_C1–C2, respectively. A (*) marks where there is a significant difference (*p*<0.01) in the AA counts between the Archaea and the *Thermotoga*. A (#) marks where there is a significant difference between the extant *Thermotoga* sequences and the reconstructed *Thermotoga* sequences. This shows there is no significant difference between the *Thermococcus* extant sequences and the *Thermococcus* ASR sequences, see Table S4 for *p*-values.

One might argue that our reconstructed *Thermotoga* MIPS has characteristics of its archaeal donor, not its new *Thermotoga* host. That would be the case if the extant genes descended from the newly arrived gene before it ameliorated to its new *Thermotoga* host. To support our argument that this is not the case, we compared the sequences of our reconstructed MIPS proteins with those of extant *Thermotoga* and *Thermococcus* MIPS sequences to look for evidence for the bacterial rather than archaeal nature of our reconstructed *Thermotoga* MIPSs. When we compared the amino acid compositions of the extant *Thermococcus* and *Thermotoga* sequences we found that amino acids H, E, D, N, T, Y, A, and G showed significantly different abundances (*p*<0.01; Figure 2 and Table S4). Using these differences, we can distinguish an archaeal from a *Thermotoga* MIPS sequence. We found that the amino acid composition of the ancestral *Thermotoga* MIPSs are slightly different from that of the average of the extant *Thermotoga* MIPSs, but is even more different from the average *Thermococcus* MIPS (Table S4). This demonstrates that our reconstructed *Thermotoga* MIPSs are representative of the *Thermotoga* host-ameliorated MIPS sequence and so reflect the thermal characteristics of that host rather than the donor.

We used similar comparisons to assess the changes that have taken place in each lineage. The average *Thermococcus* MIPS sequence is not significantly different from any of the reconstructed *Thermococcus* ACM MIPSs (Table S4). This suggests that the extant Thermococcales MIPSs have not changed significantly from their ancestral state. By contrast, the average *Thermotoga* MIPS showed significant differences in abundances of amino acids R, F, E, and G, and sometimes in I and T when compared with the reconstructed *Thermotoga* ATM MIPSs (Table S4). The composition of these proteins have apparently changed from their ancestral state, but not in the same manner as the *Thermococcus* sequences, so the ATM proteins are not descended directly from a *Thermococcus* donor sequence. It is not surprising that there have been minor changes in the descendants because they grow at temperatures slightly lower than their common ancestral *Thermotoga*.

In Methods, we explain our reasoning to decide which amino acid to use at sites of ambiguous residues. For example, the Bayesian and Ancescon method used to predict the sequence of the ancestral *Thermotoga* MIPS, node T, resulted in more than one residue at positions 119 and 191. To test if changing these two residues would have a significant change in the reconstructed proteins, amino acid sequences were constructed *in silico* with the ‘other’ most likely residues for position 119 and 191 (all possible sequences; 5 for each ATMs) and these derivatives were called ATM_T1-4′ and their IVYWREL values were calculated. The ATM_T1-4′ mean IVYWREL values (0.4018) was not significantly different from that of ATM_T1-4 proteins values (0.4110) (*p* = 0.07). This suggests that the changing of position 119 and 191 with the with the ‘other’ most likely residues does not cause a significant change in the proteins thermostability based on the correlation we have shown between IVYWREL values and *T_m_* for the MIPS proteins.

### Experimental Evidence Validates that Ancestral *Thermotoga* MIPSs are More Thermostable than Extant *Thermotoga* MIPSs

We showed in our computational analyses that the IVYWREL values of the MIPS proteins can be use to indicate thermostability, but high IVYWREL values can be related to factors other than thermostability [22], [23]. To verify that the reconstructed MIPSs are more thermostable than the extant proteins, extant MIPS homologs from species of *Thermotoga*, *Thermococcus* (*Tc.*), and *Pyrococcus* were tested *in vitro* for activity and thermostability, and their properties compared to those of the reconstructed proteins.

MIPS converts glucose-6-phosphate to *myo*-inositol-3-phosphate [4], [6], [7], [17]. The crystal structure of *Tt. maritima* MIPS has been determined (TM1419; Joint Center for Structural Genomics, PDB #3CIN, unpublished), but its catalytic activity has not been described. Only one thermophilic MIPS, from *Archaeoglobus fulgidus*, has had its activity characterized *in vitro* [24]. *A. fulgidus* MIPS is a class II aldolase [24]. Phylogenetically the *Thermotoga* MIPSs are more closely related to class I than class II enzymes. Class I enzymes use NH_4_^+^ as an allosteric activator while class II enzymes, like that from *A. fulgidus*, use metals.

The extant and reconstructed ancestral MIPSs have similar kinetic profiles (Table 2). All MIPSs tested used NH_4_^+^ as an allosteric activator, which is a characteristic of class I aldolases [25]–[27]. The temperature optima (*T_opt_*) of the extant MIPSs were found to be near those of the OGTs of the source organisms (Table 1). The IVYWREL values of the extant proteins are correlated to the experimentally tested *T_opt_* (R^2^ = 0.91; Figure S3B) of the MIPS proteins. This, in conjunction with the above computational correlations, provides further support for using the IVYWREL values as an indicator of thermostability. The MIPSs from the archaeal branches have the highest *T_opt_*, which similar to the OGT trend (Table 1). As predicted, the four ancestral MIPSs from node T have higher *T_opt_* than those of *Tt. maritima* and *Thermotoga* sp. str. RQ2 (Table 1). The higher *T_opt_* of the ancestral *Thermotoga* MIPSs indicate that this protein may have originated from an organism that lived at a higher temperature.

**Table 2:** Kinetic properties of MIPSs with the substrate glucose-6-phosphate.

Organism	Protein	K_m_ (mM)	V_max_ (nmole s^−1^)	K_cat_/K_m_ (mM^−1^ s^−1^)
*Tt. maritima* MSB8	TM1419	0.95±0.09	5.3±0.1	33±2
*Thermotoga* sp. str. RQ2	TRQ2_1313	0.44±0.09	10.3±0.7	413±67
	ATM_T1	0.63±0.10	15.5±0.8	358±44
	ATM_T2	0.46±0.08	11.3±0.9	433±52
	ATM_T3	0.54±0.15	11.8±1.0	395±84
	ATM_T4	0.60±0.06	11.6±0.3	278±23
	ACM_C1	2.34±0.46	30.8±2.4	78±10
	ACM_C2	6.27±1.94	40.6±5.8	116±24
*Tc. kodakarensis* KOD1	TK2278	0.79±0.06	23.3±0.1	428±34
*P. furiosus* DSM 3638	PF1616	0.79±0.19	5.9±0.3	135±33
	AAM_A1	2.77±0.60	27.2±3.3	173±16
	AAM_A2	1.32±0.11	20.0±0.8	264±19

Activities of enzymes were calculated using Lineweaver-Burk plots. MIPS assays were done at each enzyme’s *T_opt_* using glucose-6-phosphate as substrate. Standard deviations were determined from at least four replicates.

The melting temperatures (*T_m_*) of several MIPSs were determined using differential scanning fluorimetry (DSF) (Table 1). The *T_m_*s were determined at different pH values, because some proteins unfold at pH 7.0 at a temperature higher than 99°C, which is the maximum temperature of the thermocycler. *T_m_*s for such extremely thermostable proteins have been determined by DSF at low pH [28]. The *T_m_* (pH 3.6–4.2) was compared to the MIPS IVYWREL and *T_opt_* values of extant MIPS and they are all correlated to each other (R^2^>0.95; Figure S3D–E). This shows that there is a correlation between *T_m_* and IVYWREL values, and *T_m_* and *T_opt_* values for the MIPSs dataset. Furthermore, a correlation was shown when the OGT was compared to *T_m_* (pH 3.6–4.2) (R^2^ = 0.64–0.88; Figure S3C).

The extant archaeal MIPSs (except *Tc. sibiricus* MM 739) have the highest *T_m_* values, which correlate with the OGTs of the source organisms (Table 1). The MIPS from *Tc. sibiricus* MM 739, the tested archaeon with the lowest OGT, also had the lowest *T_m_* (Table 1). This suggests that in *Tc. sibiricus* MM 739 the composition of the MIPS protein has adapted to the lower temperature environment in which this microbe lives. An analogous adaptation to lower temperature may have happened to the MIPS that the *Thermotoga* genus acquired through HGT. The *Tc. sibiricus* MIPS IVYWREL value was higher than that of the *Thermotoga* species tested. Although using IVYWREL values to predict the thermostability of proteins can be a powerful computational approach, this result underlines the importance in doing biochemical analyses of proteins.

*Thermotoga* sp. str. RQ2 MIPS has a higher *T_m_* than that of *Tt. maritima* MIPS, which corresponds to their IVYWREL values. The *T_m_* values at pH 7.0 of the reconstructed ancestral MIPSs ATM_T1-4 are at least 3.4°C higher than that of *Thermotoga* sp. str. RQ2 MIPS, which is the most thermostable *Thermotoga* protein tested (Table 1). The average of the ATM_T1-4 values is 5.7°C at pH 7.0, which is higher than that of the average of the extant *Thermotoga* species’ proteins (Table 1). There is a significant difference between the *T_m_* values of the extant MIPSs and reconstructed ATM_T1-4 at pH 7.0 (*p*<0.01; Table S5A). The difference in *T_m_* values between these two groups is significant at pH ranging from 3.6 to 7.0 (Table S5A). The ASR of *Thermotoga* MIPSs at node T supports the hypothesis that the OGT of the last common ancestor of the *Thermotoga* genus grew at a higher OGT. Both the higher *T_m_* and higher *T_opt_* of the reconstructed *Thermotoga* MIPSs support this hypothesis (Table 1).

*Tc. kodakarensis* KOD1 and *Pyrococcus furiosus* DSM 3638 MIPSs are extremely thermostable. Using pH 4.2, the *T_m_*s of *Tc. kodakarensis* and *P. furiosus* MIPS were determined to be greater than those of the other extant MIPSs (Table 1). ACM_C1-2 IVYWREL values (0.4188 and 0.4162, respectively) are similar to the average of the extant *Thermococcus* MIPSs at node C (0.4162). *Tc. kodakarensis* MIPS was tested because it had the highest IVYWREL value of node C, 0.4215. *Tc. kodakarensis* KOD1 MIPS has a higher *T_m_* than that of ACM_C1 and ACM_C2 when measured at both pH 3.5 and 4.2 (Table 1).

The reconstructed ancestral proteins from node A (AAM_A1-2) have higher IVYWREL values relative to the average values for all three nodes and the reconstructed ancestral proteins (Figure 1). Thus, IVYWREL values for the ancestral proteins at nodes T, C, and A were generally higher than the averages of the extant proteins at each node and the more deeply located nodes had higher IVYWREL values than the more recent nodes (A>C>T). All three nodes have statistically different IVYWREL values (Table S3). AAM_A1 and AAM_A2 were the most thermostable of all the MIPS proteins tested, including *P. furiosus* MIPS (Table 1, pH 3.5). *P. furiosus* grows at the highest OGT and contains the most thermostable extant MIPS tested. There is a significant difference between the *T_m_* values of AAM_A1–A2 group compared to PF1616, and TK2278, and ACM_C1-2 group (Table S5B). These results suggest that the ancestral *Pyrococcus* may have grown at a higher OGT than the extant species.

The data gathered here leads to a general trend, the higher the OGT of the organism the higher the MIPS protein IVYWREL, *T_opt_*, and *T_m_* values. Several comparisons were made with these variables and they all support this correlation. These results are based upon a few data points and the pattern observed between OGT and IVYWREL, *T_opt_*, and *T_m_* values has not been shown with other proteins or organism. Overall, these results indicate a correlation between MIPSs IVYWREL value, *T_opt_*, and *T_m,_* and organism’s OGT, and the correlation can be used to predict the OGT of an organism or an ancestral organism given IVYWREL or *T_m_* values.

## Conclusion

This study suggests that the *Thermotoga* and *Thermococcus* ancestors may have been capable of living in hotter environments than their descendants do now. This contention is based on the fact that the reconstructed proteins at each node are more thermostable and have higher optimal catalytic temperatures than their descendant proteins. Since the thermal characteristics of extant MIPS proteins show a correlation with the OGTs of their hosts, we surmise that the hosts of our reconstructed ancestral proteins had OGTs higher than their descendants. Additionally, we showed that the MIPS sequences have ameliorated to the *Thermotoga* genome based on reconstruction of proteins from the most recent common ancestor of *Thermotoga*, which did not have the signature of the donor (Archaea) and were more similar to the extant *Thermotoga* proteins.

## Methods

### Reagents

All reagents were reagent grade and purchased from either Sigma-Aldrich Co. or Fisher Scientific, Inc., unless otherwise stated.

### Statistical Analysis

*P* values were determined either by *t*-test or *z*-test unless otherwise stated. An *f*-test was used to determine the appropriate *t*-test for a given data set. R^2^ and Pearson correlation coefficient (Pcc) were determined from a linear regression line. Standard deviations were calculated from at least three replicas. 95% confidence intervals were calculated from replicates by finding the 2.5% and 97.5% quantiles.

### Phylogenetic Analyses

Phylogenetic analyses were used to identify genes recently acquired from Archaea in the *Thermotoga*. The species of *Thermotoga* screened were *Tt. maritima* MSB8 [29], *Thermotoga* sp. str. RQ2 [29], *Tt. naphthophila* RKU-10 [30], *Tt. neapolitana* DSM 4359 [31], and *Tt. petrophila* RKU-1 [30]. In this work, the genus *Thermotoga* only refers to the above species and their close relatives as measured by 16S rRNA gene sequence comparisons. *Thermotoga lettingae and Thermotoga thermarum* were not considered as true members of the genus *Thermotoga* since their 16S rRNA gene sequence identities are too dissimilar from the true *Thermotoga* species. Genes unique to sequenced *Thermotoga* and *Thermosipho* species were identified using bidirectional BLAST and BranchClust [32]. Protein sequences were aligned using default parameters in MUSCLE v3.8.31 [33]. After visual inspection of the alignments, maximum likelihood trees were made using PhyML 3.01 with Gamma+I WAG substitution model [34]. These ML trees were used to identify genes acquired from Archaea through HGT. One of the proteins identified with a clear phylogenetic history of transfer from the Archaea to the *Thermotoga* genus was *myo*-inositol-3-phosphate synthase (MIPS).

Bayesian analyses of protein sequences were also used to screen for Archaea-derived genes using MrBayes [35]. The best model for amino acid substitution was determined using ProtTest [36]. IVYWREL values were calculated using modified scripts initially developed by Olga Zhaxybayeva (Dartmouth College).

### Tests of Models of Ancestral Reconstruction

The sequences of ancestor proteins at nodes of the MIPS phylogenetic tree were derived from analyses of extant MIPS protein sequences. The complete MIPS sequences were obtained from published genome sequences at NCBI. The partial *Thermotoga* MIPS sequences indicated below were obtained from published data [18]. *Tt. maritima* MSB8 and *Thermotoga* sp. str. RQ2 MIPS peptide sequences are 382 amino acids in length. The MIPSs partial peptide sequences from *Thermotoga* sp. str. KOL6, *Thermotoga* sp. str. RQ7, *Tt. neapolitana* LA10, *T. maritima* SL7, and *Tt. maritima* FjSS3B1 are missing 13 residues at their N-termini, and 37 residues at their C-termini. PCR amplified MIPS genes from other *Thermotoga* strains were also included in this analysis (Table S1). The full peptide sequences for these MIPSs were determined, except for that of *Thermotoga* sp. NTLA3, which is missing 67 residues at its N-terminus and 31 residues at its C-terminus. The accession numbers for these sequences are in Table S1. PhyML 3.01 with Gamma+I WAG substitution model [34] was used to construct a maximum likelihood tree using these sequences (Figure 1).

The best model for amino acid substitution was determined using ProtTest [36]. Ancescon [19] and MrBayes [37] were used to reconstruct the ancestral *Thermotoga* MIPS sequences *in silico*. Four different ASR methods were tested. First, a homogeneous substitution model was tested using Ancescon. Ancescon was run with the default parameters with optimize alpha (O) and reconstruct sequence for biological root and all internal nodes (R).

Second, a non-homogeneous substitution model was implemented to predict the ancestral proteins. ProtTest [36] was used to determine which of 112 homogeneous substitution models best fit the data. LG+G best described our dataset based on a Bayesian Information Criterion score. The Bio++ suite of programs was then used with the best model to implement the model non-homogeneously [20] by dividing the dataset *a priori* into two clades: and the *Thermotoga* species, defined as node T and its descendants (Figure 1), and the *Thermococcus* and *Pyrococcus* species, defined as all other nodes on the tree. Each clade was described by a separate set of equilibrium frequencies, while the parameters of the gamma distribution remained constant throughout the tree. Using this non-homogeneous implementation of LG+G model, the branch length and parameter values were optimized in BppML [20] using four rate categories and an initial alpha of 1. The root was placed in the *Pyrococcus* group basal to the gene transfer from *Thermococcus* to *Thermotoga*. Ancestral reconstruction was run in BppAncestor [20] using the optimized tree and parameters. The positions of gaps were inferred for reconstructed sequences using the method detailed in [38]. Briefly, to calculate the position of gaps in the ancestral sequences, we changed all of the gaps in existing sequences to Cs, and all of the amino acids to A’s. We then used the F84 substitution model in BppAncestor to determine ancestral ‘sequences,’ which represent the position of gaps as Cs. We used BppAncestor to calculate the position of gaps (Cs) in the output ancestral sequences and inserted the gaps into the actual reconstructed ancestral sequences using in-house PERL scripts (Figure S4). Alternative rootings of the tree within the *Methanococcus, Pyrococcus, Thermococcus* or *Thermotoga* did not affect ancestral sequence composition at the ancestral *Thermotoga* node.

Third, three ancestral nodes with strong bootstrap support from the PhyML tree were used for ASR of the node closest to the *Thermotoga* (node T), a *Thermococcus* node (node C) and ancestral to both node T and C and to other Archaea. MrBayes was used to construct a million trees, Burnin was used to remove the first 250,000 trees, and one tree for every 750 trees (total of 1,000 trees) was used to construct the ancestral protein for nodes A, C, and T using Ancescon. Ancescon was run with the default parameters with optimize alpha (O) and reconstruct sequence for biological root and all internal nodes (R). The probability of each residue was calculated from the 1,000 trees and averaged.

Fourth, it has been suggested that ancestral reconstruction using maximum likelihood may lead to a basis towards more thermostable protein for some datasets [21]. The Bayesian approach employed here samples from the posterior distribution to estimate the ancestral amino acid composition. Such a method is not prone to the tendency of ML methods to reconstruct ancestral proteins with a higher thermostability than they actually had [21]. This was implemented in BppAncestor, and was done using both the non-homogeneous model described above, and a homogeneous implementation of that same model. We created 1000 replicates for each ancestral sequence, determined by sampling from the posterior distribution. The mean and confidence interval of the IVYWREL biases for the ancestral sequences were calculated from these replicates (Table S2). The values within the 95% confidence interval are never more than a 5% deviation from the mean value, indicating that our dataset is not prone to the bias mentioned by [21].

### Reconstruction of Ancestral Sequences

The ancestor reconstruction methods did not assign each residue unambiguously. At positions for which more than one amino acid could be used, we used the following methods to decide which amino acid to incorporate into our reconstructed proteins. The decision process is described in detail for determining the amino acid sequence of the node T proteins and this same process was used for the proteins at nodes A and C.

The ancestor protein sequences for node T derived from the phylogenetic analyses contained a small number of gaps. Gaps that occurred in the ancestral sequence caused by a residue from the deep branching *Pyrococcus* species (the N-terminus *Pyrococcus* species had up to three residues before the methionine in the *Thermotoga* sequences) and gaps that occurred due to a residue found in only one peptide sequence were removed from the ancestral sequence. No other gaps were present for the sequence for node T. Residues assigned a probability score of 0.9000 or above were considered strongly supported and were used in the construction of the ancestral proteins. For node T, 89 residues had values below 0.9000 and were examined by other criteria to decide which amino acid should be at those positions. If two possible residues at a position had the same charge and polarity and their predictions added up to at least 0.9000 with at least one of them at or above 0.7000, the residue with the highest probability was chosen.

Of the remaining 43 residues, Ancescon predicted a probability of ≥0.7000 for 39 of them. Thirty-eight of these positions had a single amino acid in all or all but one of the extant *Thermotoga* MIPSs. That amino acid was assigned to those positions, leaving only five ambiguous residues at positions 119, 125, 126, 142, and 191.

All *Thermotoga* MIPS have Arg or Thr at position 119. When there is an Arg at 119 there is a Glu at 118, but when there is a Thr at 119 there is an Asp at 118 (Figure S5). The only *Thermotoga* to have an Arg at 119 and an Asp at 118 is *Thermotoga* sp. str. KOL6. Since the support for residue 118 being Glu is high (0.9606), Arg was placed at residue 119 in the ancestral protein.

Amino acid 125 was predicted to be either Ser or Thr, while the residue at 126 was predicted to be either Glu or Lys. In extant MIPS proteins, a Ser at 125 is associated with Lys at 126, but when there is a Thr at 125 there is a Glu at 126 (Figure S5). The only exception to this patter is *Thermotoga* sp. str. KOL6 that has a Thr at position 125 and a Lys at 126. This pattern suggests a relationship between amino acids at positions 125 and 126 that is related to the structure or function of MIPS. Consequently, two variations of the ancestral MIPS were constructed with Ser/Lys or Thr/Glu at positions 125/126, respectively.

At position 142, Lys (polar, positive; higher support) and Ile (nonpolar, neutral; lower support) were predicted. The most likely residue at this position could not be resolved, so both possibilities were constructed.

The residue at position 191 (Figure S5) was predicted to be Ile, Phe, or Tyr. Ile and Phe are both nonpolar and neutral while Tyr is polar and neutral. When the *Thermotoga* MIPSs have Ile or Phe at residue 191, they have Asn (polar and neutral) at residue 177 (Figure S5). When the *Thermotoga* MIPS have a Tyr (polar, neutral residue) at 191, all have Lys at 177 residue (polar, positive), except one has Ser (polar and neutral). The support for residue 177 being Lys is high (0.9986), so Tyr was placed at residue 191 in the ancestral protein.

Based on the above analyses, four probable ancestor proteins were predicted at node T. These reconstructed proteins were called ancestral *Thermotoga* MIPS, ATM, and labeled with their respective node and number, ATM_T1-4.

The same strategy used to predict the ancestor proteins at node T was done for those at nodes C and A. After using this analytical approach, only one residue remained questionable at node C and one at node A. At node C, position 365, Arg (polar, positive; higher support) and Gln (polar, neutral; lower support) were predicted. At node A, position 76, Glu (polar, negative) and Lys (polar, positive) were predicted. The most likely residue at these positions for nodes C and A could not be resolved, so both possibilities were constructed at each node. The reconstructed proteins for node C were called ancestral *Thermococcus* MIPS, ACM (ACM_C1-2). The reconstructed proteins for node A were called ancestral Archaea MIPS, AAM (AAM_A1-2).

### Amino Acid Composition Comparison of Archaea and Bacteria MIPS

It has been predicted that the archaeal and bacterial kingdoms use different amino acids for thermal adaptation of proteins; specifically, Gln, Ile, and positively charged amino acids [39]. In order to compare amino acid compositions between the *Thermotoga* and archaeal sequences amino acid counts were preformed for each amino acid in each sequence. All *Thermococcus* and *Methanococcus* MIPS sequences were used to determine the average Archaea counts (Figure 1). The average amino acid compositions for the extant archaeal sequences and the extant *Thermotoga* sequences were compared to determine if there were significant differences using a *z*-test (Table S4). The within group comparisons of the extant Archaea sequences and ASR ACM_C1–C2 sequences, and the extant *Thermotoga* sequences and ASR ATM_T1-4 sequences were tested for significant differences between the mean extant amino acid composition and the reconstructed composition using a *t*-test (Table S4).

### Cloning and Sequencing of MIPS-encoding Genes

MIPS encoding genes were amplified using polymerase chain reaction (PCR) (Tables S1 and S6) using Failsafe enzyme mix (Epicentre), and were cloned into pGEM-T using Easy Vector System (Promega) or directly cloned into pBAD TOPO® (Invitrogen). The genes were excised from the pGEM-T vector using restriction enzyme sites incorporated during PCR, and cloned into pBAD TOPO® and transformed into *Escherichia coli* TOP10 cells (Invitrogen). The DNA for the ATM_T1-4, ACM_C1-C2, and AAM_A1-A2 genes (Table S1) were synthesized by GenScript USA Inc., and were cloned like the other genes. The sequence of each gene was determined at the University of Connecticut DNA Biotechnology Facility.

### Expression and Purification of Ancestral and Extant MIPS Proteins

MIPS genes were expressed and purified from the pBAD TOPO® vector in *E. coli* TOP10 cells as described by the manufacturer (Invitrogen) with a few modifications. The cultures were grown in Bertani’s Lysogeny Broth (LB)/ampicillin (100 µg/ml) and induced on arabinose for 3–4 h at 37°C or overnight at 18°C.

The cultures were pelleted at 4,955×*g* for 30 min and washed with 500 mM NaCl, 0.5 mM dithiothreitol (DTT), and 20 mM imidazole-HCl, pH 7.8. Bacterial Protein Extraction Reagent (B-PER; Thermo Scientific) containing lysozyme and DNase I was used to resuspend the pellets as described by the manufacturer with a few modifications. The reaction mixture contained Halt™ Protease Inhibitor Cocktail EDTA-Free (10 µl/ml B-PER; Thermo Scientific) and RNase A (Qiagen; 1 ng/ml of B-PER reagent). The reaction mixture was incubated for at least 15 min at room temperature, and heat-treated at 70°C for 10 min to denature non-thermophilic proteins. The cell extracts were pelleted and the supernatants filtered through a 0.2 µm filter (Thermo Scientific). Immobilized metal ion affinity chromatography was performed with a His SpinTrap (GE Healthcare) or Ni Sepharose High Performance Resin (GE Healthcare) using the protocol of the manufacturer with the following modifications: 500 mM NaCl, 0.5 mM DTT, and 20 mM imidazole-HCl, pH 7.8 was used as wash buffer, and 500 mM NaCl, 0.5 mM DTT, and 500 mM imidazole-HCl, pH 7.8 was used as elution buffer. The protein was concentrated with an Ultra-4 10K Centrifugal Filter (Amicon), washed 3–5 times and resuspended in 500 mM NaCl, 0.5 mM DTT, and 50 mM imidazole-HCl, pH 7.8, and stored at 4°C. Protein concentrations were determined using the Bradford reagent and bovine serum albumin as the standard following the manufacturer’s instructions (Thermo Scientific).

### Differential Scanning Fluorimetry (DSF)

Differential scanning fluorimetry (DSF) was done as previously described with modifications [28]. The proteins were assayed in a final volume of 20 µl with concentration of 0.16% (vol/vol) 5,000× Sypro orange (Invitrogen), 150 mM NaCl, protein (1 µl protein/storage buffer was used for all reaction for consistency), and 20 mM citric acid-sodium phosphate buffer at pH 4.2 or 7.0. The fluorescence intensities were measured using a CFX96™ Real-Time PCR Detection System (BioRad) with excitation at 490 nm and emission at 530 nm. The samples were heated from 30–99°C with a heating rate of 0.5°C per min. All the assays were done in triplicate. The melting temperature (*T_m_*) is defined as the temperature at half the maximal fluorescence and was determined using Gnuplot with curve fitting to the Boltzmann equation with in-house scripts [40].

### *myo*-inositol-3-phosphate Synthase (MIPS) Assay

The activities of MIPSs were determined using a MIPS/malachite green assay as previously described with modifications [25]–[27]. The reaction conditions were optimized for *Thermotoga* sp. str. RQ2 MIPS and ATM_T1. These enzymes were shown to be in their initial velocities during the first 2.5 min. NH_4_^+^ increased the activity of all MIPSs tested. A typical MIPS reaction contained 15 mM NH_4_Cl, 20 mM D-glucose-6-phosphate, and 10 mM imidazole-HCl, pH 7.8 in a 50 µl volume. The reaction was preheated for 2.5 min in 0.2 ml Thermowell Gold Flat cap PCR tubes, (Corning Incorporated, Corning, NY) in an MJ-Mini thermocycler (BioRad). For temperatures over 99°C, reactions were carried out in a heating block. To minimize evaporation these tubes were covered with approximately 50 µl mineral oil and tube caps were not closed. After preincubation, 5 µl of enzyme was added, the reaction was incubated 1 min, and then placed on dry ice for at least 5 min. The amount of enzyme used for each reaction was empirically determined. The following amounts of enzyme were used in 50 µl reaction volumes: 2.5 µg TRQ2_1313, 7.4 µg TM1419, 3.0 µg ATM_T1, 2.5 µg ATM_T2, 2.5 µg ATM_T3, 3.0 µg ATM_T4, 3.0 µg TK2278, 2.5 µg PF1616, 3.0 µg ACM_C1, 3.0 µg ACM_C2, 3.0 µg AAM_A1, and 3.0 µg AAM_A2. At these concentrations of enzymes, the reactions were shown to be in their initial velocities in the first 2.5 min. To determine their optimal temperatures, the enzymes were tested for activity at different temperatures. TRQ2_1313, TM1419, ATM_T1, ATM_T2, ATM_T3, and ATM_T4 were tested at 70°C, 75°C, 78°C, 80°C, 83°C 85°C, 90°C, and 95°C. TK2278 and PF1616 were tested at 70°C, 75°C, 80°C, 85°C, 90°C, 95°C, 99°C, 105°C, and 110°C. ACM_C1, ACM_C2, AAM_A1, and AAM_A2 were tested at 85°C, 90°C, 95°C, 99°C, 105°C, 110°C 115°C, and 120°C. To the frozen reaction, 50 µl of 200 mM NaIO_4_ was added, mixed, centrifuged, and incubated at 37°C for 1 h to liberate the phosphate group from *myo*-inositol-3-phophate. The reaction mixture was placed at 4°C for at least 10 min and then mixed with 100 µl 1.5 M Na_2_SO_3_ to reduce the NaIO_4_. For tubes heated over 99°C with mineral oil, frozen reactions were thawed, 25 µl of sample were removed from the bottom of the tube and to this 25 µl of 200 mM NaIO_4_ and 50 µl 1.5 M Na_2_SO_3_ was used. From this mixture, 50 µl was added to 700 µl water, then 750 µl of malachite green reagent was added, mixed, and incubated for 10 min at room temperature. Phosphate was measured at 630 nm on an Evolution 300 UV-Vis Spectrophotometer (Thermo Scientific) using Na_2_HPO_4_ as the phosphate standard. The malachite green reagent consisted of 0.7 M HCl, 0.3 mg/ml malachite green oxalate, 2 mg/ml sodium molybdate dihydrate, and 0.5 mg/ml Triton-100. The malachite green reagent was filtered through a 0.2 µm filter (Thermo Scientific), and stored for no more than 1 month in the dark at 4°C. All MIPS/malachite green assays were done with at least four replicates.

## Supporting Information

Figure S1 **Maximum likelihood (PhyML) trees of related MIPS protein sequences.** The tree was constructed using PhyML. Support values were calculated from 100 bootstrap samples. Phylogenetically related microbes were grouped together and the major groups are shown: *Thermotoga* (red), Euryarchaeota (blue), Crenarchaeota (green), Thaumarchaeota and Korarchaeota (yellow), *Aquifex* (gray), and other Bacteria species (purple). Select bootstrap values with ≥70 bootstrap are indicated by black filled circles (•). Related groups were collapsed using FigTree (http://tree.bio.ed.ac.uk). Methanogenic Archaea are indicated with an asterisk. On the tree, two MIPS from methanogenic Archaea species are near the *Thermotoga* species, while 17 are located near the bottom of the tree. Homologs were gathered from the NCBI non-redundant database using *Tt. petrophila* RKU-1 peptide as query sequence. An E-value 1E-20 and Bit score of 100 were used as cut offs.
(PDF)

Figure S2 **The OGT and mean IVYWREL bias values (IVYWREL) were compared for several MIPS proteins.** All organisms with known OGT from Figure S1 were used. In cases where a range of OGT was reported, the average of the range was used. The average IVYWREL bias values for each OGT was calculated and the mean standard error is shown. Linear regression yields a Pearson correlation coefficient (Pcc) of 0.80.
(PDF)

Figure S3 **Comparison of extant organisms OGT and their MIPS proteins IVYWREL bias values (IVYWREL),** ***T_opt_*** **, and** ***T_m_*** **.** (**A**) The OGT of an organism was compared to the extant MIPS IVYWREL values. (**B**) The IVYWREL value was compared to extant MIPS *T_opt_*. (**C**) The OGT was compared to *T_m_* (pH 3.6–4.2). (**D**) The *T_m_* (pH 3.6–4.2) was compared to the MIPS IVYWREL of extant MIPS. (**E**) The *T_m_* (pH 3.6–4.2) was compared to the *T_opt_* of extant MIPS. The *T_m_* values of MIPSs were determined using DSF and standard deviations for MIPS *T_m_* values were determined from three replicates. The *T_opt_* values were determined using a MIPS/malachite green assay and values were determined from at least four replicates. *Tc. sibiricus* MM 739 was excluded from all analyses because its MIPS *T_opt_* had not been determined.
(PDF)

Figure S4 **In-house PERL scripts used to calculate the gaps and reconstructed ancestral sequences using** **BppAncestor.**
(TXT)

Figure S5 **Alignment of MIPS homologs.** Residues described in the text are boxed (red) and labeled. A blue line separates the sequences from *Thermotoga* species from those from the Euryarchaeota species. The alignment was done using MUSCLE v3.8.31 [33]. The *P. horikoshii* OT3 MIPS sequence (gi 14591380) was modified from that in the NCBI database by removal of amino acids 1–42 because they did not show homology to any other MIPSs suggesting a possible misannotation of its start codon.
(PDF)

Table S1 **Amplified MIPS-encoding genes.** Accession numbers are shown for genes sequenced in this study.
(DOC)

Table S2 **A comparison of** ***in silico*** **prediction of protein thermostability, IVYWREL values, by posterior sampling prediction to that of Ancescon/MrBayes prediction.**
(DOC)

Table S3 **Statistical analysis of IVYWREL values of extant and reconstructed MIPS proteins.**
(DOC)

Table S4 **Statistical analysis of the amino acid compositions of extant and reconstructed** ***Thermotoga*** **and** ***Thermococcus*** **MIPS proteins.**
(DOC)

Table S5 **Statistical analysis of** ***T_m_*** **(**°**C) values of extant and reconstructed MIPS proteins.**
(DOC)

Table S6 **Primers used for amplification of MIPS-encoding genes.**
(DOC)
