# Supplementary figures and images for: Reconstructed Ancestral Myo-Inositol-3-Phosphate Synthases Indicate That Ancestors of the Thermococcales and Thermotoga Species Were More Thermophilic than Their Descendants
Source: PLoS One. 2013 Dec 31;8(12):e84300. doi: 10.1371/journal.pone.0084300 (PMC3877268; doi:10.1371/journal.pone.0084300)

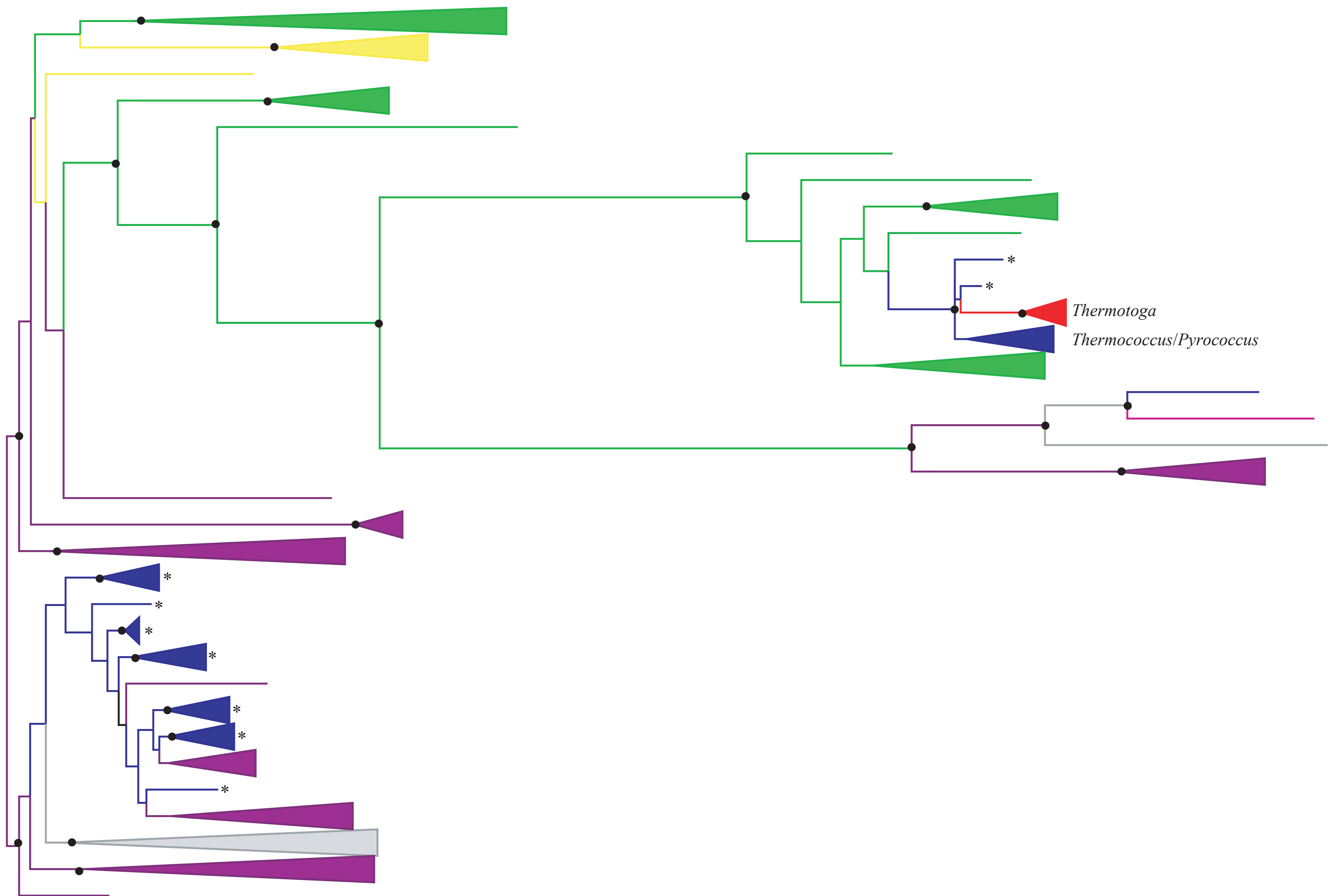

0.7

Supplement: Figure S1 — Maximum likelihood (PhyML) trees of related MIPS protein sequences. The tree was constructed using PhyML. Support values were calculated from 100 bootstrap samples. Phylogenetically related microbes were grouped together and the major groups are shown: Thermotoga (red), Euryarchaeota (blue), Crenarchaeota (green), Thaumarchaeota and Korarchaeota (yellow), Aquifex (gray), and other Bacteria species (purple). Select bootstrap values with ≥70 bootstrap are indicated by black filled circles (•). Related groups were collapsed using FigTree (http://tree.bio.ed.ac.uk). Methanogenic Archaea are indicated with an asterisk. On the tree, two MIPS from methanogenic Archaea species are near the Thermotoga species, while 17 are located near the bottom of the tree. Homologs were gathered from the NCBI non-redundant database using Tt. petrophila RKU-1 peptide as query sequence. An E-value 1E-20 and Bit score of 100 were used as cut offs. (PDF) [file pone.0084300.s001.pdf]

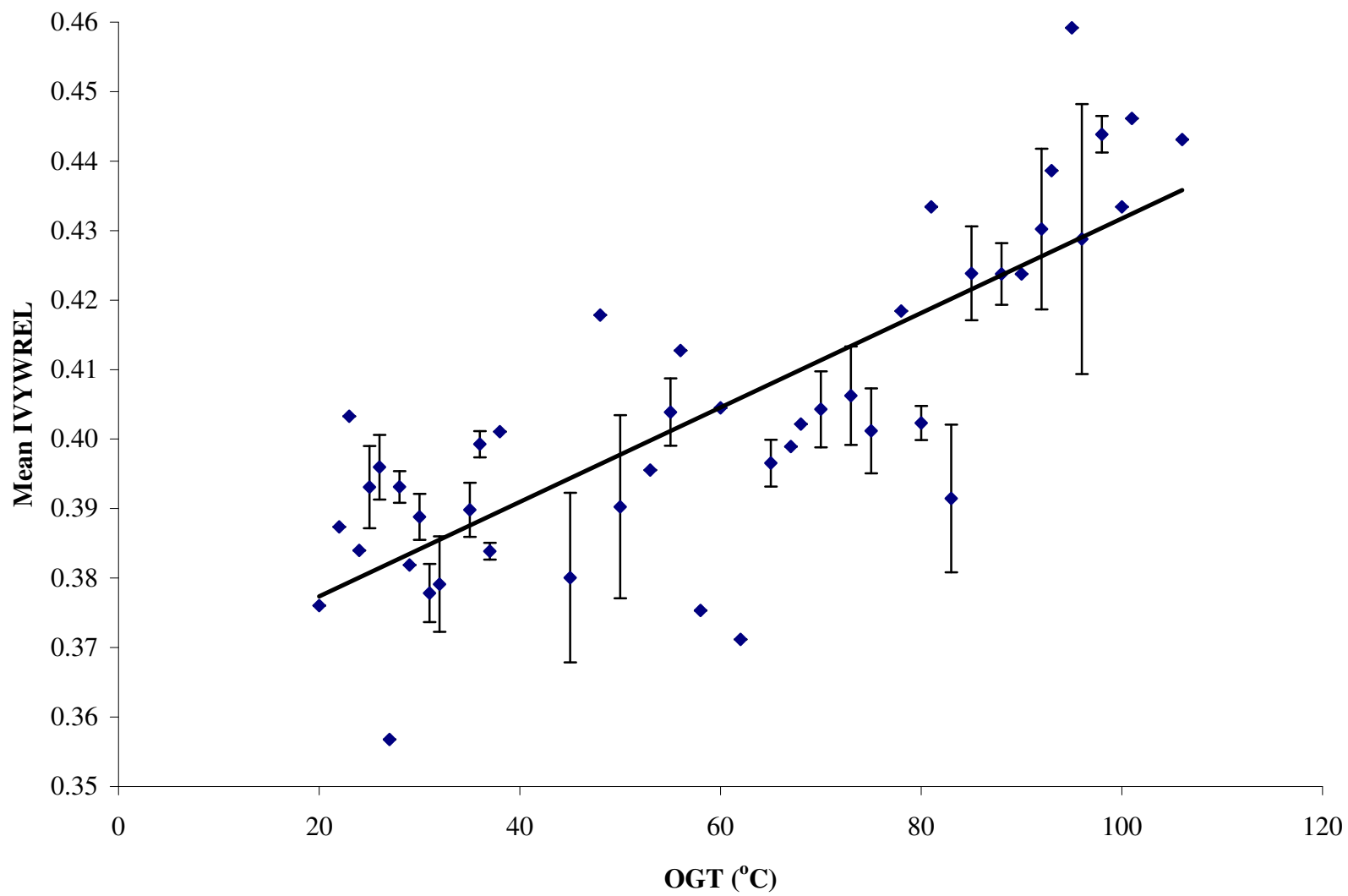

Supplement: Figure S2 — The OGT and mean IVYWREL bias values (IVYWREL) were compared for several MIPS proteins. All organisms with known OGT from Figure S1 were used. In cases where a range of OGT was reported, the average of the range was used. The average IVYWREL bias values for each OGT was calculated and the mean standard error is shown. Linear regression yields a Pearson correlation coefficient (Pcc) of 0.80. (PDF) [file pone.0084300.s002.pdf]
