# Supplementary material for: Reconstructed Ancestral Myo-Inositol-3-Phosphate Synthases Indicate That Ancestors of the Thermococcales and Thermotoga Species Were More Thermophilic than Their Descendants
Source: PLoS One. 2013 Dec 31;8(12):e84300. doi: 10.1371/journal.pone.0084300 (PMC3877268; doi:10.1371/journal.pone.0084300)
Supplement: Figure S3 — Comparison of extant organisms OGT and their MIPS proteins IVYWREL bias values (IVYWREL), Topt , and Tm . (A) The OGT of an organism was compared to the extant MIPS IVYWREL values. (B) The IVYWREL value was compared to extant MIPS Topt. (C) The OGT was compared to Tm (pH 3.6–4.2). (D) The Tm (pH 3.6–4.2) was compared to the MIPS IVYWREL of extant MIPS. (E) The Tm (pH 3.6–4.2) was compared to the Topt of extant MIPS. The Tm values of MIPSs were determined using DSF and standard deviations for MIPS Tm values were determined from three replicates. The Topt values were determined using a MIPS/malachite green assay and values were determined from at least four replicates. Tc. sibiricus MM 739 was excluded from all analyses because its MIPS Topt had not been determined. (PDF) [file pone.0084300.s003.pdf]

(A)

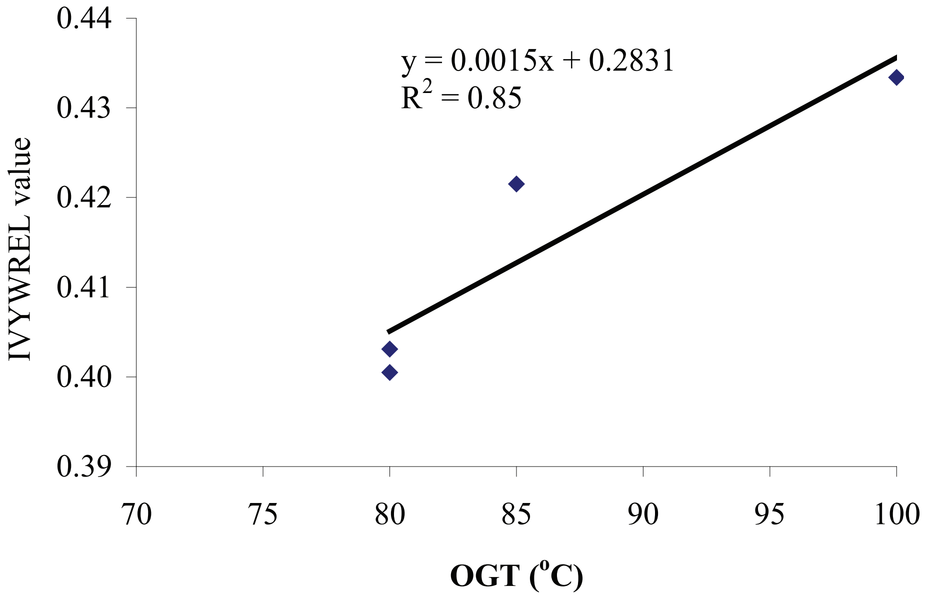

(B)

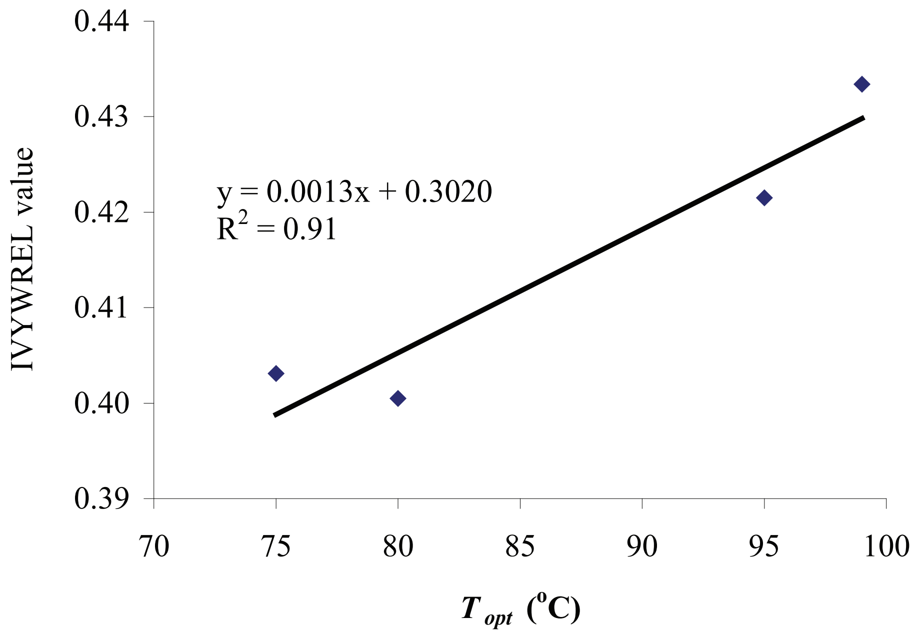

(C)

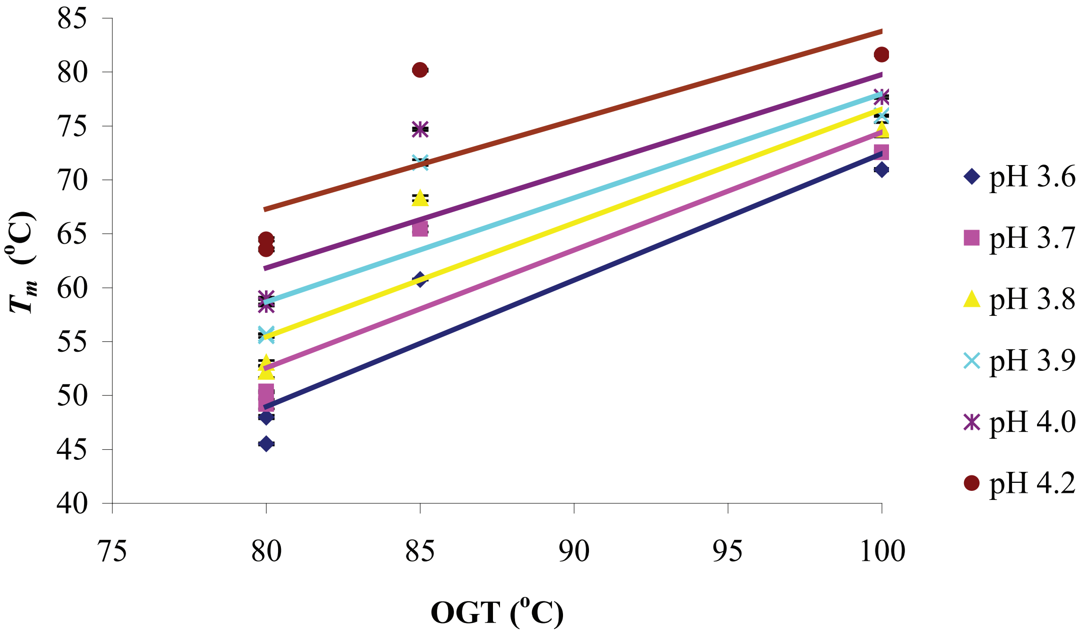

pH	Equation	R^2
3.6	y = 1.1730x - 44.8601	0.88
3.7	y = 1.0930x - 34.8780	0.81
3.8	y = 1.0548x - 28.9286	0.79
3.9	y = 0.9658x - 18.592	0.74
4.0	y = 0.8964x - 9.8658	0.70
4.2	y = 0.8246x + 1.3366	0.64

(D)

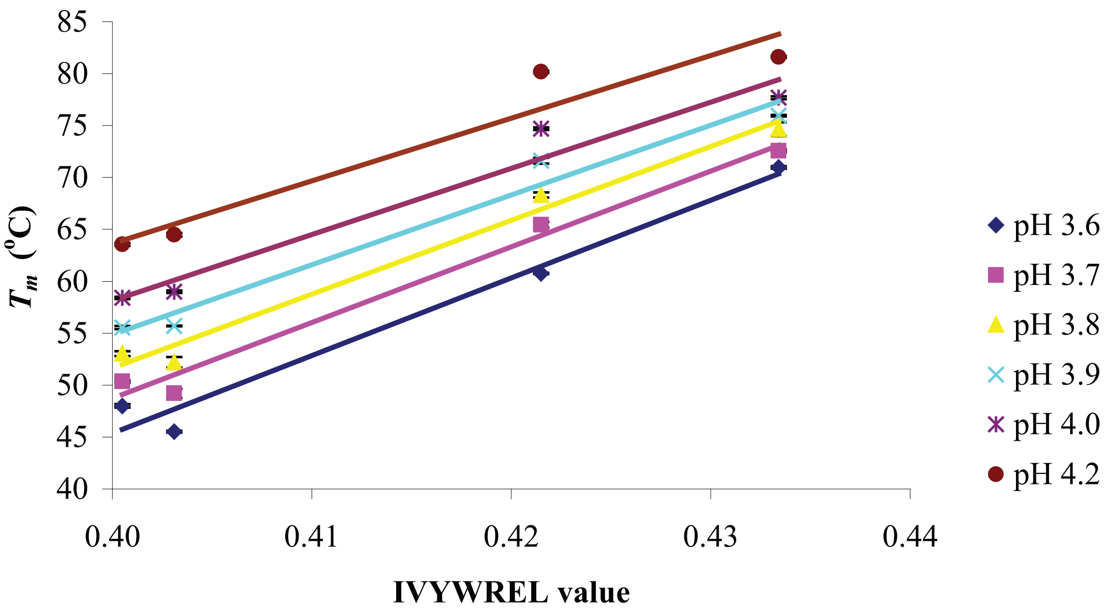

pH	Equation	R^2
3.6	y = 747.67x - 253.69	0.97
3.7	y = 729.56x - 243.1	0.98
3.8	y = 711.26x - 232.86	0.98
3.9	y = 671.71x - 213.8	0.97
4.0	y = 637.84x - 197.02	0.96
4.2	y = 604.32x - 178.11	0.93

(E)

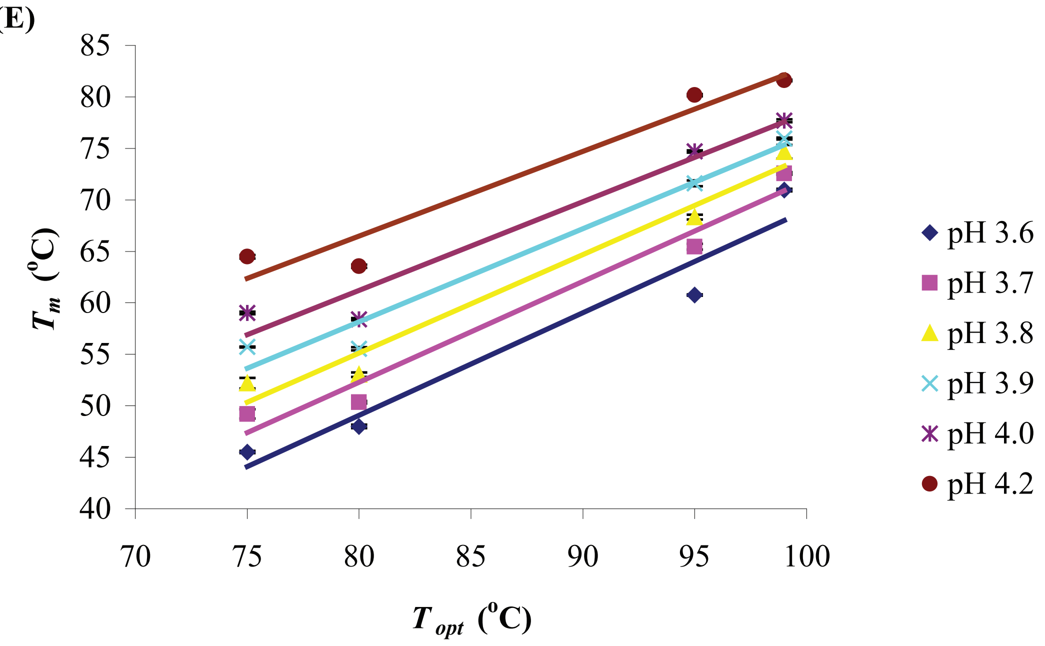

pH	Equation	R^2
3.6	y = 0.9958x - 30.577	0.95
3.7	y = 0.9784x - 25.968	0.97
3.8	y = 0.9551x - 21.286	0.97
3.9	y = 0.9041x - 14.175	0.97
4.0	y = 0.8618x - 7.742	0.96
4.2	y = 0.8222x + 0.7159	0.95
