# Supplementary material for: Reconstructed Ancestral Myo-Inositol-3-Phosphate Synthases Indicate That Ancestors of the Thermococcales and Thermotoga Species Were More Thermophilic than Their Descendants
Source: PLoS One. 2013 Dec 31;8(12):e84300. doi: 10.1371/journal.pone.0084300 (PMC3877268; doi:10.1371/journal.pone.0084300)
Supplement: Figure S5 — Alignment of MIPS homologs. Residues described in the text are boxed (red) and labeled. A blue line separates the sequences from Thermotoga species from those from the Euryarchaeota species. The alignment was done using MUSCLE v3.8.31 [33]. The P. horikoshii OT3 MIPS sequence (gi 14591380) was modified from that in the NCBI database by removal of amino acids 1–42 because they did not show homology to any other MIPSs suggesting a possible misannotation of its start codon. (PDF) [file pone.0084300.s005.pdf]

Strain	110 … 118 119 120 … 125 126 130 … 140 142 150 … 160 … 170 … 177 180 … 190 191 … 200
Thermotoga sp._SG1	SMPLKEAVERLVDEWSKLDPDVIVNTCTTEAFKPFGNKEELLKAIENNDKKRLTATQVYAYAAALYAKKRGGAAFVNVIPTYIANDPAFVEL
Thermotoga sp._SG7	SMPLKEAVERLVDEWSKLDPDVIVNTCTTEAFKPFGNKEELLKAIENNDKKRLTATQVYAYAAALYAKKRGGAAFVNVIPTYIANDPAFVGL
Tt. maritima SL7	SMTLKEAIERLVDEWSKLDPDVIVNTCTTEAFKPFGNKEELLKAIENNDKERLTATQVYAYAATLYAKKRGGAPFVNVIPTYIANDPAFVEL
Tt. neapolitana LA10	SMTLKEAIERLVDEWSKLDPDVIVNTCTTEAFKPFGNKEELLKAIENNDKERLTATQVYAYAATLYAKKRGGAAFVNVIPTYIANDPAFVEL
Tt. neapolitana DSM_435	SMTLKEAIERLVDEWSKLDPDVIVNTCTTEAFKPFGNKEELLKAIENNDKERLTATQVYAYAATLYAKKRGGAAFVNVIPTYIANDPAFVEL
Thermotoga sp._AV18	SMTLKEAIERLVDEWSKLDPDVIVNTCTTEAFKPFGNKEELLKAIENNDKERLTATQVYAYAATLYAKKRGGAAFVNVIPTYIANDPAFVEL
Thermotoga sp. RQ7	SMTLKEAIERLVDEWSKLDPDVIVNTCTTEAFRPFGNKEELLKAIEDNDKERLTATQVYAYAAALYAKKRGGAAFVNVIPTYIANDPAFVEL
Tt. maritima FjSS3B1	SMTLKEAVERLVDEWSKLDPDVIVNTCTTEAFKPFGNKKELLKAIENNDKERLTATQVYAYAAALYAKKRGGAAFVNVIPTYIANDPAFVEL
Thermotoga sp._KOL6	KMTLKEVVDRLVDEWTKLDPDVIVNTCTTEAFKPFDNKEDLMKSIENNETERLSATQVYAYAAALYASKRGGAVFVNVIPTYIANDPAFVEL
Thermotoga sp. SR1	SMTLKEAVDTLVKEWTELDPDVIVDTCTTEAFVPFGNKEDLLKAIENNDKERLTATQVYAYAAALYANKRGGAAFVNVIPTFIANDPAFVEL
Tt. maritima 2812B	SMTLKEAVDTLVKEWTELDPDVIVNTCTTEAFVPFGNKEDLLKAIENNDKERLTATQVYAYAAALYANKRGGAAFVNVIPTFIANDPAFVEL
*Tt._maritima*_MSB8	SMTLKEAVDTLVKEWTELDPDVIVNTCTTEAFVPFGNKEDLLKAIENNDKERLTATQVYAYAAALYANKRGGAAFVNVIPTFIANDPAFVEL
Tt. thermarum LA3	SMTLKEAVDTLVKEWTELDPDVIVNTCTTEAFVPFGNKEDLLKAIENNDKERLTATQVYAYAAALYANKRGGAAFVNVIPTFIANDPAFVEL
Thermotoga sp._cell2	SMTLKEAVDTLVKEWTELDPDVIVNTCTAEAFIPFGNKEDLLKAIENNDKERLTATQVYAYAAALYANKRGGAAFVNVIPTFIANDPAFVEL
Tt. petrophila RKU-1	SMTLKEAVDTLVKEWTELDPDVIVNTCTTEAFIPFGNKEDLLKAIENNDKERLTATQVYAYAAALYANKRGGAAFVNVIPTFIANDPAFVEL
Tt. naphthophila RKU-10	SMTLKEAVDTLVKEWTELDPDVIVNTCTTEAFIPFGNKEDLLKAIENNDKERLTATQVYAYAAALYANKRGGAAFVNVIPTFIANDPAFVEL
Thermotoga sp._RQ2	SMTLKEAIERLVDEWSKLDPDVIVNTCTTEAFKPFGNKEELLKAIENNDKERLTATQVYAYAATLYAKKRGGAAFVNVIPTYIANDPAFVEL
Mc. infernus ME	NLSLSEAVDKLIESWKEKGVEVIVNVCTTESFVPFNDKEELIKAIEEDKKERVTATQVYAYAACKYAKEVGGAAFINAIPTLIANDKAFVEL
*Mt._igneus*_Kol_5	EMSLKEAVEKLVEEWKELKPDVIVNTCTTEAFVPFGNKEELIKAIEENNKDRLTATQVYAYAAALYAKEVGGAAFVNTIPTLIANDPAFVEL
Tc. onnurineus NA1	EMSLKDAIEHLVNEWKELRAEVFINVCTTEAFVPFESREELEKAIEEDNRDRLTATQVYAYAIAQYAKEVGGAAFVNAIPTLIANDPVFVEL
Thermococcus sp._4557	EMTLKEAVEHLVNEWKELGAEVFINVCTTEAFQPFGSREELEKAIEEDNRDRLTATQVYAYAIAQYAKEVGGAAFVNAIPTLIANDPAFVEL
Tc. gammatolerans EJ3	EMTLSEAVEHLVSEWKELKPDVFVNVCTTEAFVPFEKKEELEKAIAEDRKDRVTATQVYVYAAAKYAKEVGGAAFVNAIPTLIANDPAFVEL
Thermococcus sp. AM4	EMTLSEAVEHLVSEWKELKAEVFVNVCTTEAFVPFEKKEELEKAIAEDRKDRLTATQVYAYAVAKYAKEVGGAAFVNAIPTLIANDPAFVEL
Tc. kodakarensis KOD1	EMTLKEAVDHLVNEWKELKPDVFVNVCTTEAFVPFESREELEKAIEENNKERLTATQFYVYAAAKYAKEVGGAAFVNAIPTLIANDPVFVEL
P. abyssi GE5	EMTLKEAVEHLVNEWKESKVEVILNVPTTEAFTPFGKLEELEKAINENNKDRLTATQVYAYAAAQYAKEVGGAAFVNAIPTLIANDPAFVEL
Pyrococcus sp. NA2	EMSLKEAIEHLVNEWKESKVEVILNVPTTESFVPFGKLEDLEKAIKEDNRDRLTATQVYAYAAAQYAKEVGGAAFVNAIPTLIANDPAFVEL
P. horikoshii OT3	EMPLKEAVEQLVSEWKDAKVEVILNVPTTESFVPFEKLENLEKAIKEDNRDRLTATQVYAYAAAQYAKEVGGAAFVNAIPTSIANDPAFVEL
P. furiosus DSM_3638	EMTLKEAIERLVEEWKEKKVDVIINVPTTEAFTPFGKLEELEKAIKDNNKERLTATQAYAYAAAQYAKEVGGAAFVNAIPTLIANDPAFVEL
P. yayanosii CH1	EMSLRDAIEHLVSEWKELKPDVIVNVPTTEAFVPFEDLEKLEKAIKEDDRDRLTATQVYAYAAAQYAKEVGGAAFVNAIPTLIANDPAFVEL
Tc. barophilus MP	RMTLKEAVEKIVEEWKELKPDVIVNVCTTEAFKPFNSKEELIKAIENDNRERLTATQVYAYAASLYAKEVGGAAFVNAIPTLIANDNAFLEL
*Tc._litoralis*_DSM_5473	EMSLKEAVEKLVEEWKKLDVDVIVNVCTTEAFVPFGNKEELIKAIENDERERLTATQVYAYAAALYAKERGGAAFVNAIPTLIANDPAFVEL
*Tc._sibiricus*_MM_739	EMTLKEAVEKLVEEWKKSTPDVIINVCTTESFTPFGNKEKLIKAIEDNNKERLTASQVYAYAAALYAKEVGGAAFVNAIPTLIANDPAFVEL
