# Supplementary material for: Reconstructed Ancestral Myo-Inositol-3-Phosphate Synthases Indicate That Ancestors of the Thermococcales and Thermotoga Species Were More Thermophilic than Their Descendants
Source: PLoS One. 2013 Dec 31;8(12):e84300. doi: 10.1371/journal.pone.0084300 (PMC3877268; doi:10.1371/journal.pone.0084300)
Supplement: Table S1 — Amplified MIPS-encoding genes. Accession numbers are shown for genes sequenced in this study. (DOC) [file pone.0084300.s006.doc]

**Table S1. Amplified MIPS-encoding genes.** Accession numbers are shown for genes sequenced in this study.

| **Organism** | **Locus** | **Primers** | **Accession number** |
| --- | --- | --- | --- |
| *Thermotoga sp. NTLA3* | MIPS_LA3 | P1 and P2 | JX266154 |
| *Thermotoga* sp. str. SG1 | MIPS_TSG1 | P9 and P10 | JX266150 |
| *Thermotoga* sp. str. SG7 | MIPS_SG7 | P9 and P10 | JX266151 |
| *Thermotoga* sp. str. AV18 | MIPS_AV18 | P9 and P10 | JX266152 |
| *Thermotoga* sp. str. SR1 | MIPS_SR1 | P1 and P2 | JX266153 |
| *Tc. sibiricus* MM 739 | TSIB_1788 | P3 and P4 |  |
| *Tt. maritima* MSB8 | TM1419 | P1 and P2 |  |
| *Thermotoga* sp. str. RQ2 | TRQ2_1313 | P1 and P2 |  |
| *Tc. kodakarensis* KOD1* | TK2278 | P5 and P6 |  |
| *P. furiosus* DSM 3638 | PF1616 | P7 and P8 |  |
|  | ATM_T1 |  | JX266146 |
|  | ATM_T2 |  | JX266147 |
|  | ATM_T3 |  | JX266148 |
|  | ATM_T4 |  | JX266149 |
|  | ACM_C1 |  | KC010430 |
|  | ACM_C2 |  | KC010431 |
|  | AAM_A1 |  | KC010432 |
|  | AAM_A2 |  | KC010433 |

*Gift from Takaaki Sato
