# Supplementary material for: Reconstructed Ancestral Myo-Inositol-3-Phosphate Synthases Indicate That Ancestors of the Thermococcales and Thermotoga Species Were More Thermophilic than Their Descendants
Source: PLoS One. 2013 Dec 31;8(12):e84300. doi: 10.1371/journal.pone.0084300 (PMC3877268; doi:10.1371/journal.pone.0084300)
Supplement: Table S2 — A comparison of in silico prediction of protein thermostability, IVYWREL values, by posterior sampling prediction to that of Ancescon/MrBayes prediction. (DOC) [file pone.0084300.s007.doc]

**Table S2. A comparison of *in silico* prediction of protein thermostability, IVYWREL values, by posterior sampling prediction to that of Ancescon/MrBayes prediction.**

| **Sequence** | **IVYWREL** |
| --- | --- |
| *Thermotoga* node T |  |
| Average extant proteins | 0.4003 |
| Posterior Sampling Prediction | 0.4085 ± 0.0051 |
| ATM_T1 | 0.4084 |
| ATM_T2 | 0.4110 |
| ATM_T3 | 0.4110 |
| ATM_T4 | 0.4136 |
|  |  |
| *Thermococcus* node C |  |
| Average extant proteins | 0.4162 |
| Posterior Sampling Prediction | 0.4168 ± 0.0073 |
| ACM_C1 | 0.4188 |
| ACM_C2 | 0.4162 |
|  |  |
| Archaea node A |  |
| Average extant proteins | 0.4416 |
| Posterior Sampling Prediction | 0.4501 ± 0.0095 |
| AAM_A1 | 0.4491 |
| AAM_A2 | 0.4465 |
