# Supplementary material for: Reconstructed Ancestral Myo-Inositol-3-Phosphate Synthases Indicate That Ancestors of the Thermococcales and Thermotoga Species Were More Thermophilic than Their Descendants
Source: PLoS One. 2013 Dec 31;8(12):e84300. doi: 10.1371/journal.pone.0084300 (PMC3877268; doi:10.1371/journal.pone.0084300)
Supplement: Table S3 — Statistical analysis of IVYWREL values of extant and reconstructed MIPS proteins. (DOC) [file pone.0084300.s008.doc]

**Table S3. Statistical analysis of IVYWREL values of extant and reconstructed MIPS proteins.** Statistical test scores, *p*-values, are shown for comparisons between groups of sequences. IVYWREL values were calculated from all fully sequenced MIPS proteins from organisms with known OGT from Note T, Node C, and for all *Pyrococcus* species (*Pyrococcus* group) from Figure 1. An *f*-test was used to determine the appropriate *t*-test for each compassion made. ND, not determined.

|  | **Node T** | **ATM_T1-T2** | **Node C** | **ACM_C1-C2** | ***Pyrococcus* group** | **AAM_A1-A2** |
| --- | --- | --- | --- | --- | --- | --- |
| **Node T** |  |  |  |  |  |  |
| **ATM_T1-4** | < 0.001 |  |  |  |  |  |
| **Node C** | < 0.001 | 0.057 |  |  |  |  |
| **ACM_C1-2** | < 0.001 | 0.022 | 0.706 |  |  |  |
| ***Pyrococcus* group** | <0.001 | < 0.001 | <0.001 | **0.003** |  |  |
| **AAM_A1-2** | < 0.001 | < 0.001 | < 0.001 | ND | 0.229 |  |
