# Supplementary material for: Reconstructed Ancestral Myo-Inositol-3-Phosphate Synthases Indicate That Ancestors of the Thermococcales and Thermotoga Species Were More Thermophilic than Their Descendants
Source: PLoS One. 2013 Dec 31;8(12):e84300. doi: 10.1371/journal.pone.0084300 (PMC3877268; doi:10.1371/journal.pone.0084300)
Supplement: Table S4 — Statistical analysis of the amino acid compositions of extant and reconstructed Thermotoga and Thermococcus MIPS proteins. (DOC) [file pone.0084300.s009.doc]

**Table S4. Statistical analysis of the amino acid compositions of extant and reconstructed *Thermotoga* and *Thermococcus* MIPS proteins.** Statistical test scores, *p* values, are shown for comparisons between groups of sequences. The *Thermococcus* vs. *Thermotoga* row shows the comparison between the average amino acid compositions for the extant *Thermococcus* MIPSs and the extant *Thermotoga* MIPSs, which were compared to determine if there were significant differences using a *z*-test. The ACM_C1-C2 and upper group of ATM_T1_1_-T4_1_ rows show tests for significant differences between the mean amino acid composition of extant MIPSs of their respective lineages and the reconstructed MIPSs compositions using a *t*-test. The lower group of ATM_T1_2_-T4_2_ rows shows tests for significant differences between the extant *Thermococcus* MIPSs and the ASR *Thermotoga* sequences using a *t*-test.

| Comparison | Sequence | R | K | C | S | Q | P | L | M | F | V | W | I | **H** | **E** | **D** | **N** | **T** | **Y** | **A** | **G** |
| --- | --- | --- | --- | --- | --- | --- | --- | --- | --- | --- | --- | --- | --- | --- | --- | --- | --- | --- | --- | --- | --- |
| *Thermotoga* vs *Thermococcus* | Averages of extant MIPS | 0.14 | 0.05 | NA | 0.10 | 0.03 | 0.19 | 0.05 | 0.01 | 0.03 | 0.26 | NA | 0.25 | **<0.01** | **<0.01** | **<0.01** | **<0.01** | **<0.01** | **<0.01** | **<0.01** | **<0.01** |
| Average *Thermococcus* MIPS vs ACM | ACM_C1 | 0.02 | 0.16 | NA | 0.34 | 0.07 | 0.34 | 0.16 | 0.50 | 0.16 | 0.41 | NA | 0.03 | 0.16 | 0.27 | 0.38 | 0.28 | 0.21 | 0.50 | 0.22 | 0.01 |
|  | ACM_C2 | 0.16 | 0.16 | NA | 0.34 | 0.03 | 0.34 | 0.16 | 0.50 | 0.16 | 0.41 | NA | 0.03 | 0.16 | 0.27 | 0.38 | 0.28 | 0.21 | 0.50 | 0.22 | 0.01 |
| Average *Thermotoga* MIPS vs ATM | ATM_T1_1_ | **<0.01** | NA | 0.08 | 0.22 | NA | 0.08 | NA | 0.04 | **<0.01** | 0.14 | NA | 0.22 | NA | **<0.01** | 0.04 | 0.36 | **<0.01** | 0.28 | 0.04 | **<0.01** |
|  | ATM_T2_1_ | **<0.01** | NA | 0.08 | 0.22 | NA | 0.08 | NA | 0.04 | **<0.01** | 0.14 | NA | **<0.01** | NA | **<0.01** | 0.04 | 0.36 | **<0.01** | 0.28 | 0.04 | **<0.01** |
|  | ATM_T3_1_ | **<0.01** | NA | 0.08 | 0.01 | NA | 0.08 | NA | 0.04 | **<0.01** | 0.14 | NA | 0.22 | NA | **<0.01** | 0.04 | 0.36 | 0.17 | 0.28 | 0.04 | **<0.01** |
|  | ATM_T4_1_ | **<0.01** | NA | 0.08 | 0.01 | NA | 0.08 | NA | 0.04 | **<0.01** | 0.14 | NA | **<0.01** | NA | **<0.01** | 0.04 | 0.36 | 0.17 | 0.28 | 0.04 | **<0.01** |
| Average *Thermococcus* MIPS vs ATM | ATM_T1_2_ | 0.16 | 0.02 | NA | 0.34 | 0.07 | 0.34 | 0.08 | 0.01 | 0.16 | 0.19 | NA | 0.19 | **<0.01** | **<0.01** | **<0.01** | **<0.01** | **<0.01** | **<0.01** | **<0.01** | **<0.01** |
|  | ATM_T2_2_ | 0.16 | 0.05 | NA | 0.34 | 0.07 | 0.34 | 0.08 | 0.01 | 0.16 | 0.19 | NA | 0.27 | **<0.01** | **<0.01** | **<0.01** | **<0.01** | **<0.01** | **<0.01** | **<0.01** | **<0.01** |
|  | ATM_T3_2_ | 0.16 | 0.05 | NA | 0.07 | 0.07 | 0.34 | 0.08 | 0.01 | 0.16 | 0.19 | NA | 0.19 | **<0.01** | **<0.01** | **<0.01** | **<0.01** | **<0.01** | **<0.01** | **<0.01** | **<0.01** |
|  | ATM_T4_2_ | 0.16 | 0.16 | NA | 0.07 | 0.07 | 0.34 | 0.08 | 0.01 | 0.16 | 0.19 | NA | 0.27 | **<0.01** | **<0.01** | **<0.01** | **<0.01** | **<0.01** | **<0.01** | **<0.01** | **<0.01** |
