# Supplementary material for: Reconstructed Ancestral Myo-Inositol-3-Phosphate Synthases Indicate That Ancestors of the Thermococcales and Thermotoga Species Were More Thermophilic than Their Descendants
Source: PLoS One. 2013 Dec 31;8(12):e84300. doi: 10.1371/journal.pone.0084300 (PMC3877268; doi:10.1371/journal.pone.0084300)
Supplement: Table S5 — Statistical analysis of Tm (°C) values of extant and reconstructed MIPS proteins. (DOC) [file pone.0084300.s010.doc]

**Table S5. Statistical analysis of *T_m_* (°C) values of extant and reconstructed MIPS proteins.** Statistical test scores, *p*-values, are shown for comparisons between groups of sequences. **(A)** The *T_m_* of proteins at different pH from the *Thermotoga* group, consists of the TM1419 (*Tt. maritima* MSB8 MIPS) and TRQ2_1313 (*Thermotoga* sp. str. RQ2 MIPS), were compared to the ATM_T1-4 group. **(B)** At pH 3.5, the *T_m_* of ATM_T1-4 group were compared to PF1616 (*P. furiosus* DSM 3638 MIPS) and to TK2278 (*Tc. kodakarensis* KOD1 MIPS), and to the ACM_C1-2 group. The *T_m_* values of MIPSs were determined using DSF from three replicates. An *f*-test was used to determine the appropriate *t*-test for each compassion made.

| **(A)** |  |
| --- | --- |
| **pH** | ***Thermotoga* group vs. ATM_T1-4** |
| 3.6 | < 0.001 |
| 3.7 | < 0.001 |
| 3.8 | < 0.001 |
| 3.9 | < 0.001 |
| 4.0 | < 0.001 |
| 4.2 | < 0.001 |
| 4.4 | < 0.001 |
| 4.7 | < 0.001 |
| 4.8 | < 0.001 |
| 4.9 | < 0.001 |
| 5.0 | < 0.001 |
| 5.1 | < 0.001 |
| 5.3 | < 0.001 |
| 5.4 | < 0.001 |
| 5.6 | < 0.001 |
| 5.7 | < 0.001 |
| 5.8 | < 0.001 |
| 5.9 | < 0.001 |
| 6.0 | < 0.001 |
| 6.1 | < 0.001 |
| 6.2 | < 0.001 |
| 6.3 | < 0.001 |
| 6.4 | < 0.001 |
| 6.5 | < 0.001 |
| 6.6 | 0.001 |
| 6.7 | < 0.001 |
| 6.8 | 0.001 |
| 6.9 | 0.001 |
| 7.0 | 0.002 |

| **(B)** |  |  |  |
| --- | --- | --- | --- |
| **pH 3.5** | **PF1616** | **TK2278** | **ACM_C1-2** |
| AAM_A1-2 | < 0.001 | < 0.001 | < 0.001 |
