# Supplementary material for: Reconstructed Ancestral Myo-Inositol-3-Phosphate Synthases Indicate That Ancestors of the Thermococcales and Thermotoga Species Were More Thermophilic than Their Descendants
Source: PLoS One. 2013 Dec 31;8(12):e84300. doi: 10.1371/journal.pone.0084300 (PMC3877268; doi:10.1371/journal.pone.0084300)
Supplement: Table S6 — Primers used for amplification of MIPS-encoding genes. (DOC) [file pone.0084300.s011.doc]

**Table S6. Primers used for amplification of MIPS-encoding genes.**

| **Primer** | **Cloning primers sequence (5’ to 3’)** |
| --- | --- |
| P1 | CATATGGTCAAGGTCCTGATCCTCGG |
| P2 | GGATCCTTATTACAGCCACTTCGGTTTCA |
| P3 | CCATGGTCATACTAGGTCAGGGATATGTGG |
| P4 | GTTTAAACTTATTAATGGTGATGGTGATGATGAAACCATTTTGGCTTTA |
| P5 | CATATGGTGAGGGTTGTCATACTCGGACAG |
| P6 | GGATCCTTATTAGAGCCACCTGGGCTTCAGC |
| P7 | ATTATTGGTTAGGGTAGCAATTATAGGC |
| P8 | GGATCCTTATTAGAGGTATCTTGGGGGTAGC |
| P9 | CATATGGTCAAAGTCCTGATTCTTGGT |
| P10 | GGATCCTTATTACAGCCATTTTGGTTTCA |
